# The prevalence and socio-demographic correlates of depressive symptoms among Cypriot university students: a cross-sectional descriptive co-relational study

**DOI:** 10.1186/s12888-014-0235-6

**Published:** 2014-08-20

**Authors:** Sokratis Sokratous, Anastasios Merkouris, Nicos Middleton, Maria Karanikola

**Affiliations:** Department of Nursing, Faculty of Health Sciences, Cyprus University of Technology, Vragadinou Street, Limassol, Cyprus

**Keywords:** Depression, Psychological distress, Psychiatric symptoms, Young adults, Cyprus, Students, Center for Epidemiology Studies- Depression Scale (CES-D), Parental characteristics, Health related behaviors, Clinical symptoms

## Abstract

**Background:**

Previous findings in the literature suggest that the occurrence of depressive symptoms among university students is associated with specific socio-demographic characteristics. No related research studies have been conducted among university students in Cyprus. The current study aims to add more evidence to the literature by estimating the prevalence of clinical depressive symptoms and their association with individual, parental, academic and health-related behavior characteristics.

**Methods:**

A descriptive cross sectional study with internal comparison was performed. The occurrence of depressive symptoms was assessed by the Center for Epidemiology Studies - Depression Scale (CES-D). Clinical depressive symptoms were reported as CES-D values ≥ 20. The socio-demographic and other characteristics of the participants were assessed using a questionnaire specifically designed for the present study. Both questionnaires were completed anonymously and voluntarily by 1,500 students (29.9% males and 70.1% females, response rate 85%).

**Results:**

The prevalence of clinical depressive symptoms [CES-D score ≥ 20] was 27.9%. Among other, strong positive associations with clinical depressive symptoms were observed with a) positive personal and family history of depression (OR 2.85, 95% CI: 1.77 – 4.60), b) self -assessed poor physical and mental health (OR 11.30, 95% CI: 7.05 – 18.08). Moreover, students with learning disabilities, as well as those who were dissatisfied with the major under study, the quality of the educational system, the living arrangement, their social life and the available university facilities (OR 2.73, 95% CI: 2.00 – 3.72) were more likely to report clinical depressive symptoms.

**Conclusions:**

The results of the present study highlight specific individual, parental, academic and health-related behavior characteristics of the students associated with the presence of depressive symptoms. Thus, targeted interventions considering the socio-demographic profile of vulnerable students for early recognition and manifestation of mental health disturbances may be designed. Moreover, the relatively high prevalence of clinical symptoms of depression within this particular cultural context may warrant further investigation in longitudinal studies.

## Background

Depression in young adults is a serious public health problem, and at the same time a source of immense human suffering [1]. With regard to student populations, it is recognized as a common and debilitating disturbance, whilst relative symptoms are evident in all areas of functioning, including motivation, concentration, perception of self-worth and mood [2]. Moreover, depressive symptoms may not only disrupt students’ academic performance and social functioning, but may also be associated with tobacco, alcohol and drug abuse [3]. Furthermore, according to international data, it is evident that depression may impact students of any age, gender, ethnicity and socio economic status [4].

The prevalence of depressive symptoms among university students is relatively high, estimated between 10.2% [5] and as high as 71.2% [6]. Severe depressive symptoms range between 2.3% and 10.9% in this population [7,8]. The wide range of estimates in the prevalence of depressive symptoms may, to some extent, be the result of the diverse a) methodological approaches, b) psychometric properties of the tools used, and c) socio-cultural characteristics of the different target populations. Additionally, regarding the aforementioned fluctuation in the prevalence of depressive symptoms among students, one has to consider the multi-factorial aetiology of mental disorders, including genetical predisposition [9,10], socio-demographic variables [4-6,11-13], or early life experiences and stressful life events [2].

With regard to the socio-demographic characteristics of the students reporting depressive symptoms, a wealth of published evidence exists s [4-8,11-17]. These data show that certain factors (e.g. being female, family history of mental disorders or depression) are strongly related to the experience of depressive symptoms compared to other [4]. However, there is a number of inconsistencies in the manner that socio- demographic characteristics are addressed in published studies [4]. For example, commonly researchers focus on the most frequently studied demographic factors, i.e. gender or positive family history of mental disorders, without explaining the reasons why they have excluded others [4,6]. Furthermore, factors related to students’ family status, health-related behaviors, subject of studies and academic profile are underrepresented in the literature [4].

Data revealing specific socio-demographic factors associated with the manifestation of depressive symptoms among students may be useful in identifying vulnerable groups of young adult [1-3]. Moreover, targeted interventions may be formed in order to assist in both early identification as well as address the specific characteristics of these groups [18]. There is complete lack of studies investigating the prevalence of depressive symptoms and possible associations with socio-demographic variables in Greek-Cypriot university students. Other than providing an estimate for the frequency of the disturbance, such as study will further out understanding on potential associations with related factors within this particular cultural context [19].

### Aim

The aim of the present study is to investigate a) the prevalence of clinical depressive symptoms, and b) possible associations between clinical depressive symptoms and i) individual, ii) parental, iii) academic characteristics, as well as iv) health status and v) substance abuse related variables.

## Methods

### Study population and design

A descriptive cross-sectional study with internal comparisons was performed between November 2010 and May 2011 amongst all undergraduate students of the Cyprus University of Technology (CUT), one of three public universities in the Republic of Cyprus. CUT is the second largest university with 2,452 active students across 10 Departments in 5 Faculties, offering access to a free education via national examinations.

The study was approved by the National Bioethics Committee as well as the Ethics Committee of the University. Prior to data collection, the Heads of all Departments were informed about the purpose of the study and the data collection procedures, securing their consent. All active undergraduate students (N 1,783 at the time of the study) were eligible to participate, independent of age, gender and nationality. Students enrolled in postgraduate programs of study, doctoral programs or students enrolled in other short-term educational programs were excluded.

The questionnaire pack along with an information sheet explaining the purpose of the study was distributed to the students during class time (either in lecture theatres, classrooms, or labs) and verbal consent was obtained. Participation in the study was voluntary and anonymous in order to guarantee confidentiality. After a short briefing, any students who did not wish to participate had the opportunity to leave the classroom. The final sample consisted of 1.500 undergraduate students (response rate of 85%). With regard to the 283 non-participants, 240 were absent on the day of the survey, 20 declined participation and 23 were excluded from the analysis due to missing/incomplete data. The questionnaires were returned in a collection box in sealed envelopes. The research team coordinated the data collection with the Studies and Student Affairs Office of the University in order to ensure that it would not coincide with mid-terms, final examinations or any other potentially stressful study-related activities, such as hospital or industry placements, internships etc.

### Instruments

#### Socio-demographic data questionnaire

The socio-demographic and other characteristics (e.g. academic profile) of the sample were assessed using a questionnaire specifically designed for the present study. This included ***individual*** (age, gender, residency, ethnicity, marital status, number of children, living status during study and employment status), ***parental*** (parental marital status, loss of parent (s), parents’ level of education, parents’ employment status, and annual family income), ***academic*** (academic year of study, faculty, whether faculty of study was student’s first choice, academic performance, failure to complete a course, learning disabilities, level of satisfaction with the program of study, quality of education system, living arrangement, social life and of available university facilities), ***health status*** (chronic physical disorder or disability, mental health problems, positive family history of mental health disorders and depression, level of self- assessed physical and mental health status), **and*substance abuse related behavior*** (use of tobacco, number of tobacco cigarettes smoked, alcohol consumption, use of legal and illegal drugs) variables.

#### Center for Epidemiology Studies - Depression Scale (CES-D)

The prevalence of depressive symptoms was assessed with the use of the Center for Epidemiology Studies - Depression Scale (CES-D). The CES-D was developed by Radloff [20] as a screen tool to measure the severity of symptoms of depression in community populations. The scale consists of 20 items. Responders are asked to rate each item on a scale from 0 to 3, on the basis of ‘how often have you felt this way during the past week’, 0 rarely or none of the time (less than 1 day), 1 some or a little of the time (1 - 2 days), 2 occasionally or a moderate amount of time (3 - 4 days), and 3 most or all of the time (5 - 7 days). The items include statements about depressive mood, perception of worthlessness and feelings of hopelessness, loss of appetite, poor concentration and sleep disturbances. The scale does not include items for increased appetite or duration of sleep, anhedonia, psychomotor agitation or retardation, guilt, or suicidal thoughts [20]. Four of the items are worded in a positive direction to control for response bias. The CES-D scores range from 0 to 60. Higher scores indicate more severe depressive symptoms. A score of 16 or higher has been used extensively as the cut-off point for clinical depressive symptoms [20]. However, false positives in the order of 15 - 20% have resulted from the use of this cut-off point, leading some researchers to suggest that a higher cut-off point be used [21]. In primary care settings a cut-off value of 20–22 on the CES-D is commonly used to increase its specificity [22]. For the purposes of the present study, we aimed to investigate the degree of depressive symptoms with values greater than 20 in the CES-D scale, thus reporting the prevalence of clinical depressive symptoms.

Internal consistency reliability as measured by Cronbach’s alpha has been found to be approximately 0.85 in community samples and 0.90 in patient samples [20]. Split-half reliability ranges from 0.76 to 0.85 [20]. In the present study, the CES-D scale was administrated in the Greek language, which is the native language of the vast majority of the students attending public universities in Cyprus. Although this scale has been validated for the Greek language in previous studies [23], it has not been used in the Cypriot population before, and particularly among student populations. As a result, the psychometric properties of the scale were tested. In particular, Cronbach’s alpha coefficient for internal consistency was 0.90 and Guttman split-half alpha was 0.89. Cronbach’s alpha values for the subscales were 0.90 for depressed mood, 0.89 for psychomotor and somatic symptoms, 0.90 for reduce positive affect, and 0.90 for interpersonal difficulties. The average three-week test-retest reliability coefficient for the CES-D total score was 0.73.

### Data analysis

The Statistical Package for Social Sciences Software (SPSS - version 17) was used to analyse the data. Descriptive statistics for all socio-demographic and other variables were calculated, expressed as appropriately in frequencies, mean values and standard deviation. The CES-D item scores were summed up to provide an overall score, theoretically ranging from 0 to 60, after reversing positively phrased items 4, 8, 12, and 16. Unless more than 5 items on the scale were missing (in which case a score is generally not calculated), in all other cases, the overall score was the sum of the items for which a response was provided divided by the number of answered items. The associations between the occurrence of depressive symptoms and each of the socio-demographic characteristics under study were investigated using chi-square tests. For all statistical tests, p values of 0.05 or lower were considered statistically significant. In parallel, odds ratio (and 95% confidence intervals) of depressive symptoms for each of the socio-demographics characteristics were estimated in logistic regression models. Backward stepwise multivariable logistic regression models were used in order to select among the large number of variables considered the final set associated with depressive symptoms controlling for the potential confounding effect of the rest of the variables in the final model. Unlike a previous analysis of the data focusing on the association between stressful life events and depressive symptoms [19], the aim here was not to test certain variables based on a priori defined hypothesis. Since the intention was to select the set of variables among the over 30 (some of which are highly intercorrelated) that best describe an increased odds of depressive symptoms in University student population, use of a backward technique was deemed appropriate. For all statistical tests in multivariable logistic regression model, p values of 0.10 or lower were considered statistically significant.

## Results

### The socio-demographics characteristics of the sample and prevalence of clinical depressive symptoms

The final sample consisted of 1,500 respondents (response rate 85%) from all 10 Departments of the University, of whom 448 were male (29.9%) and 1,052 (70.1%) were female. The mean age of the participants was 20.3 years (SD 2.1, range: 18–40). Table 1 presents the basic socio-demographic characteristics of the participants, along with the prevalence estimates for clinical depressive symptoms. Most students were living in metropolitan areas (Ν 800, 58%), whilst 380 (27.6%) resided in suburban areas and 198 (14.4%) in rural areas. The vast majority were of Cypriot origin (N 1,415, 94.3%), 67 students (N 67, 4.5%) were Greek and only 18 students (N 18, 1.2%) were from elsewhere. Only 6.2% (N 92) of the participants were married or living with a partner, and 0.4% (N 6) of them were divorced. A significant proportion of students were employed (N 437, 29%) during the semester. Nearly half of the participants (43.9%) were students in the Faculty of Health Sciences, and specifically in the Department of Nursing, which is by far the largest Department of the University. The minimum and maximum CES-D scores were 0 and 57 (scale range: 0 – 60). The mean value was 15.7 with a standard deviation of 10.6. However, almost one in three students (N 419, 27.9%) scored 20 or higher on the CES-D scale.

**Table 1:** **Prevalence of clinical depressive symptoms (CES-D ≥ 20) by classification of participants in terms of basic socio-demographic characteristics**

	**TOTAL**		**Students without clinical depressive symptoms**		**Students with clinically significant depressive symptoms**		**X2**	**DF**	**P value**
	**N**	**%**	**N**	**%**	**Ν**	**%**			
**Age**							0.10	2	0.947
*17-20*	933	62.2	672	72.0	261	28.0			
*21-24*	502	33.5	361	71.9	141	28.1			
*25-40*	65	4.3	48	73.8	17	26.2			
**Gender**							4.11	1	0.042
*Male*	448	29.9	339	75.7	109	24.3			
*Female*	1052	70.1	742	70.5	310	29.5			
**Ethnicity**							1.12	2	0.570
*Cypriot*	1415	94.3	1024	72.4	391	27.6			
*Greek*	67	4.5	45	67.2	22	32.8			
*Other*	18	1.2	12	66.7	6	33.3			
**Residency (longest)**							6.76	2	0.034
*Metropolitan area*	817	54.5	608	74.4	209	25.6			
*Rural areas*	250	16.4	170	59.7	80	40.3			
*Sub-urban areas*	433	29.1	303	66.9	130	34.1			
**Marital status**							0.11	2	0.945
*Single*	1402	93.5	1010	72.0	392	28.0			
*Married/Living with a person*	92	6.1	67	72.8	25	27.2			
*Separated/divorced*	6	0.4	4	66.7	2	33.3			
**Children**							1.37	4	0.242
*No*	1468	97.9	1055	71.9	413	28.1			
*Yes*	32	2.1	26	81.2	6	18.8			
Living status during study							2.75	1	0.030
*Alone*	333	22.2	228	68.5	105	31.5			
*Cohabiting*	1167	87.8	853	73.1	314	26.9			
**Employment Status**							1.31	1	0.250
*Unemployed*	1067	71.1	778	72.9	289	27.1			
*Employed*	433	28.9	303	70.0	130	30.0			

### Differences in the frequency of clinical depressive symptoms in relation to students’ individual characteristics

With regard to individual characteristics, statistically significant differences in the prevalence of clinical depressive symptoms were noted by gender, with higher frequency among female students (29.5% vs. 24, 3%, p 0.042). Similarly, those residing in rural areas presented higher prevalence of clinical depressive symptoms compared to students living in sub-urban or metropolitan areas (40.3% vs. 34.1% and 25.6%, p 0.034, respectively). In addition, students who were living alone reported higher prevalence of clinical depressive symptoms (31.5% vs. 26.9%, p 0.030) (Table 1).

### Differences between groups of the occurrence of clinical depressive symptoms in relation to students’ parental characteristics

With regard to students’ family characteristics, it was observed that the prevalence of clinical depressive symptoms was higher among students whose parents were divorced (35.7% vs. 26.7%, p 0.007). Similarly, students who reported loss of at least one parent also presented higher frequency of clinical depressive symptoms (39.7% vs. 23.7%, p 0.017), as well as those from higher educational attainment family backgrounds (i.e. 35.5% vs. 30.5 vs. 23.4, p < 0.001 among those whose father was a University graduate compared to lower educational attainment. Similarly, students whose father was unemployed reported higher occurrence of clinical depressive symptoms (34.0 vs. 26.9, p < 0.035 respectively) (Table 2).

**Table 2:** **Prevalence of clinical depressive symptoms (CES-D ≥ 20) by classification of participants in terms of the parental characteristics**

	**TOTAL**		**Students without clinical depressive symptoms**		**Students with clinically significant depressive symptoms**		**X2**	**DF**	**P value**
	**Ν**	**%**	**Ν**	**%**	**Ν**	**%**			
**Parental Marital Status**							7.40	1	0.007
*Together*	1287	85.5	944	73,3	343	26,7			
*Divorced*	213	14.2	137	64.3	76	35.7			
**Loss of parent(s)**							5.70	1	0.017
*Yes*	78	5.2	47	62.3	31	39.7			
*No*	1422	94.8	1034	72.7	388	27.3			
**Mother’s educational level**							1.67	2	0.433
Uneducated/primary/secondary	419	27.9	298	71.1	121	28.9			
*Further education/college*	783	52.2	575	73.4	208	26.6			
*University*	298	19.9	208	69.8	90	30.2			
**Father’s educational level**							16.42	2	<0.001
Uneducated/primary/secondary	518	34.5	360	69.5	158	30.5			
Further education/college	726	48.4	556	76.6	170	23.4			
*University*	256	17.1	165	64.5	91	35.5			
**Mother’s employment status**							2.52	1	0.112
*Unemployed*	226	84.9	928	72.8	346	27.2			
*Employed*	1274	15.1	153	67.7	73	32.3			
**Father’s employment status**							4.45	1	0.035
*Unemployed*	212	14.1	140	66.0	72	34.0			
*Employed*	1288	85.9	941	73.1	347	26.9			
**Annual family income**							1.54	3	0.673
*0-19500*	736	49.1	522	70.9	214	29.1			
*19501-28000*	423	28.2	309	73.0	114	27.0			
*28001-36300*	198	13.2	142	71.7	56	27.3			
*>36301*	143	9.5	108	75.5	35	24.5			

### Differences between groups of the occurrence of clinical depressive symptoms in relation to students’ academic characteristics

A statistically significant difference in the prevalence of depressive symptoms was noted with regard to the subject of studies. In particular, students from the faculty of Engineering and Technology reported the higher occurrence of clinical depressive symptoms (35.1%, p 0.001). In addition, associations were observed with the level of academic performance. Students who failed to pass or complete a course reported higher prevalence of clinical depressive symptoms compared to those students who successfully completed the requirements within each semester (35.1% vs. 26.3%, p 0.003). Students who achieved significantly lower grades than their peers presented the highest frequency of clinical depressive symptoms (50%, p 0.045). Additionally, higher prevalence of symptoms was observed among students with learning disabilities (43.9% vs. 23.6%, p < 0.001), those whose subject of studies was not their first choice (30.4% vs. 25.5%, p < 0.036), those reporting low satisfaction with their studies (49.7% vs. 22.2%, p < 0.001) or the quality of education received (43.7% vs. 21.1%, p < 0.001).

The level of satisfaction with living arrangements, social life and the quality of available university facilities was also associated with the occurrence of clinical depressive symptoms. Those with the lowest levels of satisfaction reported higher prevalence of clinical depressive symptoms (51.5% vs. 14.1%, p < 0.001). Interestingly, there was no statistically significant difference on the occurrence of depressive symptoms with regard to the academic year of study (Table 3).

**Table 3:** **Prevalence of clinical depressive symptoms (CES-D ≥ 20) by classification of participants in terms of the academic characteristics**

	**TOTAL**		**Students without clinical depressive symptoms**		**Students with clinically significant depressive symptoms**		**X** ^**2**^	**DF**	**P value**
	**Ν**	**%**	**Ν**	**%**	**Ν**	**%**			
**Academic year of study**							1.82	3	0.610
*First*	443	29.5	326	73.6	117	26.4			
*Second*	427	28.4	297	69.7	129	30.3			
*Third*	375	25.1	275	72.9	102	27.1			
*Fourth*	255	16.9	183	72.0	71	28.0			
**Faculty**							18.21	4	0.001
*Geotechnical sciences and Environmental management*	164	10.9	128	78.0	36	22.0			
*Management and Economics*	168	11.2	116	69.0	52	31.0			
*Applied Arts Communication*	219	14.6	48	67.6	71	32.4			
*Engineering and Technology*	291	19.4	189	64.9	102	35.1			
*Health Sciences*	658	43.9	500	76.0	158	24.0			
**First choice of study in present school/faculty**							4.40	1	0.036
*Yes*	749	49.9	558	74.5	191	25.5			
*No*	751	51.1	523	69.6	228	30.4			
**Academic performance (on the basis of marks)**							9.73	4	0.045
*9-10*	89	5.9	62	69.7	27	30.3			
*7-8*	931	62.1	685	73.6	246	26.4			
*6-5*	447	29.9	317	70.9	130	29.1			
*<5*	33	2.1	17	50.0	16	50.0			
**Failure to pass/complete unit within a course**							8.95	1	0.003
*Yes*	285	19.0	185	64.9	100	35.1			
*No*	121	81.0	896	77.7	319	26.3			
**Learning difficulties**							51.22	1	<0.001
*Yes*	319	21.3	179	56.1	140	43.9			
*No*	1181	78.7	902	76.4	279	23.6			
**Level of satisfaction with program/course of study**							92.54	1	<0.001
*No/low*	320	20.8	156	50.3	164	49.7			
High*/very* high	1180	79.2	925	77.8	155	22.2			
**Level of satisfaction with quality of the education system**							91.34	1	<0.001
*No/low*	497	33.1	280	56.3	217	43.7			
High*/very* high	1003	66.9	801	79.9	202	21.1			
**Level of satisfaction with living arrangement, social life and quality of available university facilities**							243.70	1	<0.001
*No/low*	945	63.0	812	49.5	133	51.5			
High*/very* high	555	37.0	269	85.9	289	14.1			

### Differences between groups of the occurrence of clinical depressive symptoms in relation to students’ self-reported health and health related behaviors

The prevalence of clinical depressive symptoms was higher among those participants who reported the presence of a chronic physical disorder/disability (35.7% vs. 27.2%, p 0.041) or a mental health problem (53.7 vs. 25.4%, p <0.001). Moreover, participants who self-assessed their physical (61.3 vs. 19.2%, p <0.001) or mental (73.1 vs. 12.8%, p <0.001) health as poor or very poor during the last month reported higher prevalence of clinical depressive symptoms compared to those who self assessed them as very good or excellent. As expected, students who had been admitted in a psychiatric clinic or had received a treatment for a severe mental health problem reported the highest frequency of clinical depressive symptoms. In addition to personal history, the prevalence of clinical depressive symptom was higher among students with a positive family history of mental health disorders (35.9 vs. 26.6%, p <0.006) (Table 4).

**Table 4:** **Prevalence of clinical depressive symptoms (CES-D ≥ 20) by classification of participants in terms of self-reported health and health-behaviors**

	**TOTAL**		**Students without clinical depressive symptoms**		**Students with clinically significant depressive symptoms**		**X2**	**DF**	**P value**
	**Ν**	**%**	**Ν**	**%**	**Ν**	**%**			
**Chronic physical disorder or disability**							4.18	1	0.041
*No*	1371	94.1	998	72.8	373	27.2			
*Yes*	129	8.6	83	63.3	46	35.7			
**Mental health problem (e.g. depression)**							48.64	1	<0.001
*No*	1366	1.1	1019	74.6	347	25.4			
*Yes*	134	8.9	62	46.3	72	53.7			
**Admission to mental health hospital/clinic**							10.34	1	< 0.001
*No*	1496	99.3	1081	72,3	415	27.7			
*Yes*	4	0.7	0	0.00	4	100.0			
**Physical health self-assessment during last month**							128.0	2	<0.001
Excellent/very good	1000	66.7	808	80.8	192	19.2			
*Good*	407	27.1	237	58.2	170	41.8			
*Poor/very poor*	93	6.2	36	38.7	57	61.3			
**Mental health self-assessment during last month**							323.10	2	<0.001
*Excellent/very good*	919	61.3	801	87.7	118	12.8			
*Good*	410	27.3	234	57.1	176	42.9			
Poor/very poor	171	11.4	46	26.9	125	73.1			
**Family history of mental health disorders (e.g. depression)**							7.63	1	0.006
*No*	1291	86.1	947	73.4	344	26.6			
*Yes*	209	13.9	134	64.1	75	35.9			

Table 5 presents the prevalence of depressive symptoms in relation to tobacco and alcohol consumption, as well as to drugs misuse. The prevalence of clinical depressive symptoms was statistically significantly higher in smokers than in non smokers (25.2% vs. 71.2%, p 0.002). Moreover, there appeared to be a stepwise increase in the prevalence of clinical depressive symptoms in terms of smoking habits(e.g. 25% among non-smokers, 30-50% among occasional smokers, 58.1% among those who report smoking often but not daily vs. 71.2% among daily smokers). A similar pattern was observed in terms of the reported number of cigarettes smoked per day. No statistically significant difference in the prevalence of clinical depressive symptoms was observed in terms of alcohol consumption. Finally, in terms of drug misuse, only 4.2% of the students reported using them even occasionally. While there was an indication of a stepwise increase in the prevalence of clinical depressive symptoms in terms of the frequency of drug use, inferences are limited by the very small sample sizes (Table 5).

**Table 5:** **Prevalence of clinical depressive symptoms (CES-D ≥ 20) by classification of participants in terms of substance use behaviors**

	**TOTAL**		**Students without clinical depressive symptoms**		**Students with clinical depressive symptoms**		**X2**	**DF**	**P value**
	**Ν**	**%**	**Ν**	**%**	**Ν**	**%**			
**Smoking habit**							18.95	2	0.002
*Never*	986	66.3	737	74.8	249	25.2			
*Rare (once a month)*	75	5.1	46	61.3	29	38.7			
*Occasionally (once week)*	61	4.1	37	60.7	24	39.3			
*Often (2–3 times per week)*	54	3.6	35	64.8	19	35.2			
*Very often (3–5 times per week)*	27	1.7	14	51.9	13	58.1			
*Daily (5–7 times per week)*	297	19.3	85	29.8	212	71.2			
**Number of cigarettes**							13.41	3	0.004
*None*	986	69.3	773	74.8	248	25.2			
*1-10*	236	15.8	161	67.4	75	32.6			
*11-20*	77	5.1	56	72.7	21	27.3			
*21-60*	201	9.7	91	63.7	73	36.3			
**Alcohol consumption**							4.03	5	0.544
*Never*	412	27.3	292	70.9	120	29.1			
*Rare(once a month)*	496	33.3	357	71.0	139	28.0			
*Occasionally (once week)*	414	27.6	310	74.9	104	25.1			
*Often (2–3 times per week)*	126	8.3	89	70.6	37	29.4			
*Very often (3–5 times per week)*	42	2.8	27	64.3	15	35.7			
*Daily (5–7 times per week)*	10	0.7	6	60.0	4	40.0			
**Number of drinks**							0.70	3	0.873
*No alcohol consumption*	412	41.1	284	70.6	120	29.4			
*1-2*	600	33.3	435	72.7	165	27.3			
*3-4*	358	17.3	261	72.9	97	27.1			
*5-16*	130	8.3	93	71.5	37	28.5			
**Drugs addiction**							11.98	5	0.035
*No drugs use*	1435	95.8	1042	72.7	393	27.3			
*Rare (once a month)*	45	2.9	27	64.1	17	38.6			
*Occasionally (once week)*	6	0.5	4	66.7	2	33.3			
*Often (2–3 times per week)*	6	0.4	3	50.0	3	50.0			
*Very often (3–5 times per week)*	5	0.2	1	20.0	4	80.0			
*Daily (5–7 times per week)*	3	0.2	0	0,0	3	100.0			
**Type of substance used**							8.59	2	0.014
*No drugs used*	1435	95.8	1042	72.7	394	27.3			
*Light drugs*	61	4.1	37	61.6	24	38.4			
*Hard drugs*	4	0.1	0	0.00	4	100.0			

### Multivariable backward stepwise logistic regression results

The results of the backward stepwise logistic regression analysis are presented in Table 6. In the multivariable analysis, some of the strongest associations were observed with positive personal (OR 1.88, 95% CI: 1.80 – 4.62) or family history of mental health disorder (OR 2.85, 95% CI: 1.77 – 4.60). In addition, students who self-assessed either their physical (OR 1.45, 95% CI: 0.79 – 2.52) or mental health during last month (OR 11.30, 95% CI: 7.05 – 18.08) as poor were more likely to report clinical depressive symptoms. Independent associations in the magnitude of 1.5- to 2-fold increase in the odds of reporting depressive symptoms were also observed by female gender (OR 1.70, 95% CI: 1.17 – 2.33), residing outside the main metropolitan areas (OR 1.60, 95% CI: 1.16-2.08), divorced or separated parental status (OR 1.60 95% CI: 2.87 – 2.91), paternal higher educational attainment (OR 1.75, 95% CI: 1.13 – 2.61). Associations of similar magnitude were observed with learning disabilities (OR 1.85, 95% CI: 1.30 – 2.87), dissatisfaction with subject of study (OR 1.95, 95% CI: 1.37 – 2.77), the quality of the educational system (OR 1.66, 95% CI: 1.21 – 2.27) and more so with dissatisfaction with living arrangement, social life and the quality of available university facilities (OR 2.73, 95% CI: 2.00 – 3.72). Finally, smoking (OR 1.40 95% CI: 1.00 – 1.89) and drug use (OR 5.44 95% CI: 2.95 – 8.84) were associated with the occurrence of clinical depression symptoms (p value <0.10, for each variable). Results were unchanged when using the forward method instead.

**Table 6:** **Adjusted odds ratios (and 95% CI) of clinical depressive symptoms (CES-D ≥ 20) by individual, paternal, academic and health behavior characteristics as estimated in multivariable backward stepwise logistic regression analysis**

**CES-C (≥20/60)**	**B**	**S.E**	**Wald**	**DF**	**Adjusted** ^**†**^		
					**OR**	**(95% CI)**	**P value**
**Gender**							
*Male*					1		-------
*Female*	0.50	0.17	8.17	1	1.70(1.17-2.33)		0.004
**Residency (longest)**							
*Metropolitan area*					1		-------
*Rural areas*/*Sub-urban areas*	0.44	0.15	8.82	1	1.60(1.16-2.08)		0.003
**Children**							
*No*					1		------
*Yes*	1.11	0.60	33.57	1	3.06(0.96-9.74)		0.050
**Parental Marital status**							
*Married*					1		--------
*Divorced*	0.43	0.25	3.10	1	1.60(0.87-2.91)		0.079
**Father’s educational level**							
Uneducated/primary/secondary				1	1		-------
*Further education/college*	0.54	0.21	6.48	1	0.70(0.51-0.97)		0.031
*University*	0.31	0.16	3.83	1	1.75(1.13-2.61)		0.011
**Faculty**							
*Geotechnical sciences and Environmental management*					1		-------
*Management and Economics*	0.41	0.32	1.70	1	1.51(0.81-2.81)		0.193
*Applied Arts Communication*	0.45	0.30	2.19	1	1.56(0.86-2.83)		0.139
*Engineering and Technology*	0.57	0.28	3.94	1	1.80(1.00-3.08)		0.047
*Health Sciences*	0.09	0.27	0.12	1	1.10(0.65-1.86)		0.724
**Learning difficulties**							
*No*					1		-------
*Yes*	0.60	0.17	12.37	1	1.85(1.30-2.57)		<0.001
**Mental health problem (e.g. depression)**							
*No*					1		-------
*Yes*	1.06	0.24	19.18	1	2.88(1.80-4.62)		<0.001
**Mental health assessment during last month**							
*Excellent/very good*					1		-------
*Good*	1.26	0.16	56.94	1	3.55 (2.55-4.90)		<0.001
*poor/very poor*	2.42	0.24	101.88	1	11.30(7.05- 18.08)		<0.001
**Physical health assessment during last month**							
Excellent/very good					1		------
*Good*	0.36	0.17	4.68	1	1.43(1.03-1.80)		0.041
*Poor/very poor*	0.47	0.29	1.37	1	1.45(0.79-2.52)		0.031
**Family history of mental health disorders (e.g. depression)**							
*No*					1		
*Yes*	1.62	0.24	18.5	1	2.85(1.77-4.60)		<0.001
**Level of satisfaction with program/course of study**							
High*/very high*					1		-------
*No/low*	0.67	0.18	14.00	1	1.95(1.37-2.77)		<0.001
**Level of satisfaction with quality of the education system**							
*High/very high*					1		------
*No/low*	0.50	0.16	9.95	1	1.66(1.21-2.27)		<0.001
**Level of satisfaction with living arrangement, social life and quality of available university facilities**							
*High/very high*					1		--------
*No/low*	1.00	0.58	40.2	1	2.73(2.00-3.72)		<0.001
**Smoking habit**							
*No*					1		-------
*Yes*	0.32	0.16	0.83	1	1.40(1.00-1.89)		0.050
**Drugs addiction**							
*No*					1		-------
*Yes*	1.63	0.28	40.6	1	5.44(2.95- 8.84)		0.002

^†^Variables included in the first stage: age, gender, residency (longest), ethnicity, family status, number of children, living status during study, employment status, academic year of study, faculty, first choice of study in school/faculty, academic performance, failure to complete/pass unit within course, learning difficulties, level of satisfaction with program/course followed, level of satisfaction with quality of the education system, level of satisfaction with living arrangement, social life and quality of available university facilities, parental marital status, loss of parent (s), mother’s educational level, father’s educational level, mother’s employment status, father’s employment status, annual family income, chronic physical disorder or disability, mental health problem -depression, physical and mental health assessment during last month, family history of mental health disorders - depression, smoking habit, number of cigarettes, alcohol consumption, number of drinks, drugs addiction.

## Discussion

### The prevalence of clinical depressive symptoms of the sample

The primary scope of the present study was to provide an estimate for the prevalence of clinical depressive symptoms among university students in Cyprus. Furthermore, the study explored clinical depressive symptoms and their association with individual, parental, academic and health-related characteristics. The study revealed that one third of students reported clinical depressive symptoms (27.9%). Moreover, the evidence identified strong links between these symptoms and specific socio-demographic, academic and health behavior characteristics.

The frequency of clinical depressive symptoms was found to be consistent with most of the studies reported by other European countries and studies from the USA. Remarkably, the frequency of clinical depressive symptoms among female students ranges from 25% to 46%, whereas lower frequency is commonly observed among male students; ranging from 12% to 34% [13,16,24,25]. The consistency of research findings could support the idea that universality does not only apply on consumed goods, but also on sentiment and general mental health status in specific populations [26].

In contrast, one might argue that this consistency might seem paradoxical due to concrete cultural differences between the aforementioned countries and Cyprus [26]. However, despite the Cypriot culture being similar to and resembling the Greek culture Papazisis et al. [27] reported a higher prevalence of depressive symptoms (52.4%) in a sample of Greek nursing students. This difference however, may stem from the fact the Papazisis et al. [27] applied a different methodological approach and instrument to assess the level of depressive symptoms. In addition the sample only comprised of nursing students. Moreover, one may argue that their results support the fact that the professional and social status of Greek nurses is in fact low [28], a factor which has been found to be associated with psychological distress [29].

In contrast, the prevalence of depressive symptoms in the present study was much higher than the study of nursing students in Sweden (10.2%) [5]. What is more, nursing students in Sweden reported the lowest prevalence of depressive symptoms, universally [26]. A possible explanation for this is that Swedish nurses perceive their professional work as respectful and fulfilling [30], which may function as a buffer system against psychological distress [28,29]. However, this research was performed over a decade ago (2002) and may not represent the current Swedish university students.

### Differences in the frequency of clinical depressive symptoms in relation to students’ individual and paternal characteristics

In this study there were significant differences in clinically significant depressive symptoms between genders. Female students reported a higher frequency (OR 1.70 95% CI: 1.17 – 2.33) and this is supported by several studies. Nevertheless, there are also a number of studies which contradict this finding or conclude no discrepancies between the two genders [9,14,31-34]. Generally, a higher prevalence of depressive symptoms amongst females has been attributed to socio-cultural factors, including factors related to gender role, as well as biological and psychological explanations [34-36]. In addition, it has also been proposed that women are generally more likely to accept and report such symptoms [4,36]. Overall, this finding is in agreement with the W.H.O’s reports that stated higher frequency of psychopathology in women [37].

Moreover, our study shows that the Cypriot students with permanent residency in rural and sub rural areas had a significantly higher risk of depression when compared to those living in metropolitan areas. Students in rural and sub rural areas are 1.60 times more likely to manifest clinically significant depressive symptoms compared with residing in metropolitan areas (OR 1.60 95% CI: 1.16-2.08). This may relate to both economic and social factors. For instance, a large proportion (23.5%) of students residing in rural areas reported poor economic family circumstances. In addition, young people living in rural areas may feel more “isolated”, receiving limited social stimuli compared to their colleagues living in metropolitan areas [26]. All the above might have an impact on the available social support, the development of coping mechanisms or to spirituality issues, and cause emotional pressure [26], which may lead to increased risk of mental disturbances [26,38].

In addition, our research reports that there is a significant connection between students with children and levels of depression. The study showed that students with children are 3 times more likely to develop depressive symptoms than those without (OR 3.06 95% CI: 0.96 – 9.74). Other research evidence supports this finding [5,26]. It is evident that being a mother, bearing the responsibilities of motherhood along with those of academic pursuits may lead to exposure to prolonged stress [5,26]. Such demands, in conjunction with other stressful life events e.g. a positive family history of mental disorder, poor physical health, drug abuse and disapproval of academic pursuits, may put students with the aforementioned characteristics at a high risk of disturbed mental status [19].

An additional risk factor revealed was the family status of the student’s parents, and in particular the case of separated or divorced parents. Specifically, students whose parents were divorced were 1.60 times more at risk of showing clinically significant depressive symptoms than students whose parents were living together (OR 1.60 95% CI: 2.87 – 2.91). This could be explained through findings which support that the availability of effective support systems functions as a buffer system against mental health problems [39]. Divorce or separation of parents can lead to a decrease in family support and may be a traumatic stress or for a child. There are studies showing that individuals who have experienced a significant loss during childhood (such as death of a loved one or parental divorce) are more likely to develop depressive symptoms during adulthood [39-45].

Interestingly it was found that students whose fathers’ have higher educational attainment (i.e. university degree) were more likely to report clinical depressive symptoms. (OR 1.75 95% CI: 1.13 – 2.61). This could derive from increased parental pressure from parents with a higher educational background [46] as the latter may have higher expectations of their children, i.e. to follow their academic footsteps and go on to occupations with higher social acknowledgement, and subsequent higher socio-economical power and status [26]. This might also explain why students from the Engineering and Technology School (commonly higher pass marks are required in the national entry exams) were found to be at a higher risk of depressive symptoms compared to students from other departments, e.g. the Nursing Department [26].

### Differences between groups of the occurrence of clinical depressive symptoms in relation to students’ academic characteristics

As mentioned above, evidence from this study suggests Engineering and Technology Cypriot students, manifest higher levels of clinically significant depressive symptoms than students studying in other faculties. Specifically in the multivariate model (Table 6), in relation to the faculty of Geotechnical Sciences and Environmental Management, only the faculty of Engineering and Technology showed a marginally statistically significant effect in comparison with the other faculties (OR 1.80, 95% CI: 1.00 - 3.08, p = 0.047). Although these differences in depression levels were not due to demographic mediator factors, such as age, gender, residency or the students’ year of study [26]; such differences might due to students varied experiences and variations in the organizational structure of each faculty [26]; and/or this most likely related to academic pressure (students in this faculty showed high level of academic performance) and social demands that college environment place on those students [19]; and/or may due to increase parental pressure from those parents with a higher educational background [46]. All the above may result in high levels of stress that eventually lead to depressive symptomatology.

Analysis of our results showed a significantly higher occurrence of clinically significant depressive symptoms amongst Cypriot students with learning difficulties. Cypriot students with learning difficulties have almost twice (OR 1.85, 95% CI: 1.30 - 2.87) to manifastate clinical depressive symptoms compared with others students without learning difficulties. In addition these students demonstrated a reduction in learning, may caused by a decrease in the level of information absorbed, and/or a decrease in their learning ability [47]. This special group seems to have particular psychological and practical difficulties, due to the peculiarities of their learning difficulties [48]; this with combination with anxiety, stress and academic failure, may probably led to academic and social isolation [49].

Moreover, remarkable findings of this study is that 1 in 3 Cypriot students whom reported with no or low level of satisfaction with their course/specialism those with no or low level of satisfaction with the quality of the education system those with no or low level of satisfaction with living arrangement, their social life and the quality of available university facilities, was up to 2.73 (no or low level of satisfaction with their course/specialism , OR 1.95 95% CI: 1.37 – 2.77, no or low level of satisfaction with the quality of the education system, OR 1.66 95% CI: 1.21 – 2.27 and no or low level of satisfaction with living arrangement, their social life and the quality of available university facilities, OR 2.73 95% CI: 2.00 – 3.72) times more at risk of manifestating clinically significant depressive symptoms compared with those whose reported with high level of satisfaction with their course/specialism, the quality of the education system and living arrangement, their social life and the quality of available university facilities. It is obvious that the quality of the relationship between students and university, affects their general, social, academic and emotional adjustment at the university [49], as well as their intention to remain and study until graduation. The interaction between the students and the university and their relation to the above, is somehow an indicator of general adjustment [50] and thus an indicator of mental health status [26].

### Differences between groups of the occurrence of clinical depressive symptoms in relation to students’ self-reported health and health related behaviors

The present study demonstrated that any physical health problems and physical disabilities were associated with depressive symptoms. Of course, physical illness is a typical symptom of depressive symptoms in students [51-53]. However, little attention has been given to this remark, because students are considered as an age group with very good physical health. One possible explanation for this could lie in the fact that students who experience physical illness have to deal both with the difficulties of daily activities and, possibly, with the social isolation coming from the combination of an introvert character and poor acceptance by fellow students.

Similarly, our results revealed a significant difference between students who self-assessed as having physical and mental health problems and those who did not. Participants who assessed their physical (OR 1.45, 95% CI: 0.79 – 2.52) or mental health (OR 11.30, 95% CI: 7.05 – 18.08) during last month as poor or very poor presented higher prevalence of clinically significant depressive symptoms compared to those students who assessed their physical health during last month as very good or excellent. In addition, an important finding in our research was that all Cypriot students (N 4) who had received treatment regarding mental health in a hospital or a clinic still showed symptoms of depression. An interesting characteristic of depression in young people is that young people who recover from a major depressive episode continue to manifest concomitant psychopathology, depressive thinking, low self-esteem, negative expectations about the future and ineffective problem solving strategies [53]. Students have a high probability of depression recurrence and of developing a new episode. In addition to these problems, students are also insensitively teased by peers because of their mental health problem, which negatively impacts on the students’ social behavior and can lead to increased introvercy.

In relation to family history of depression, in this study, the prevalence of clinically significant depressive symptoms in Cypriot students was significantly higher compared with those who had a family history depression (OR 2.85 95% CI: 1.77 – 4.60), or mental health problem (OR 1.88 95% CI: 1.80 – 4.62). Undoubtedly, the existence of a history of depression in the family is a key factor that increases the incidence of depression among Cypriot university students. It is worth noting, however, that the most common symptoms of depression in people with a family history appear in both the student and the general population. Many researches pointed out that depression was more frequent in students who had a family history of it compared with those who did not [6,25,54], and identified results similar to the present study supporting that students with a family history of depression are up to 2.5 times more likely to have depression compared with those with no family history [55]. Our results may be explained with the genetic epidemiology data suggesting that younger age of onset is associated with family history of depression [56].

With regards to drug abuse, the prevalence rates of substance use behaviors have been well documented among undergraduate students [57-60]. In regards to drug and substance use, the current research shows that smoking (OR 1.40 95% CI: 1.00 - 1.89) and drug addiction show an increased risk of manifestating clinically significant depressive symptoms (up to 5.45 times experiencing symptoms of depression for drug users, OR 5.44 95% CI: 2.95 -8.84). One explanation for this could be that since the level of stress and anxiety in Cypriot university students is rather high due to a stressful environment, a low allowance, and an intensive study tempo, they may use substances more. This is also in agreement with a study showing that smokers with anxiety disorders reported greater anxiety sensitivity, anxiety symptoms, agoraphobic avoidance, depressed mood, negative effect, stress, and life interference, when compared to non-smokers [60]. There is also some evidence that nicotine has antidepressant properties, which may explain the relationship between smokers and high occurrence of depression as people may self-medicate through smoking [61]. However, based on the cross-sectional design of the majority of such studies, including the present one, we cannot conclude whether drug abuse is a mean of self- medication against depressive symptoms, or if drug abuse leads to depressive symptoms. In any case, drug abuse seems to co-occur with depressive symptoms [60].

### Limitations

The above findings need to be viewed in the context of certain methodological limitations. The data collection took place in the students’ classrooms, hence, students that were absent on that day or students who declined to participate were not included in the analysis. While the response rate was particularly high, it is not clear whether the observed prevalence of depressive symptoms may in fact be an underestimate of the true prevalence, since it is likely that those who suffer from psychological distress or mental problems skip classes more often. More importantly, this was a descriptive cross-sectional study, precluding any inference on causality. At least with regards to some characteristics of the students reverse causality may be at play. For instance, behaviour characteristics (smoking, drugs addiction), or academic characteristics (e.g. failing a unit/course), may be the result rather than the cause of depression. Furthermore, Nowadays, depression research has given increasing consideration to the possibility of complex and reciprocal relations between stress and depression. Not only does stress increase the risk for depression (i.e. a stress exposure model of depression), but depression, or depressogenic vulnerabilities, in turn, may also increase susceptibility to stressful events that are at least in part influenced by the individual [39,62-64]. Longitudinal studies should aim to explore particular life-related factors that may lead to depressive symptoms. Finally, cross-national comparisons are difficult and there is a need for collaborative international studies to investigate the prevalence of depressive symptoms among student populations across different settings and cultures, by employing common psychometric tools and standard methodology. Nevertheless, the large sample in the present study and the use of the most appropriate and robust scale (the CES-D) to measure students’ depressive symptoms, allowed for a more accurate measurement of the manifestation of depressive symptoms and its correlation with socio-demographic characteristics. More importantly, in contrast to previous studies, the present study did not focus on the effect of certain demographic variables on depressive symptoms, but examine a large number of socio-demographic and educational variables. Additionally, it should be noted that backward techniques tend to capitalize on chance, while variables excluded from the final model are no more controlled for their possible confounding effect. Nevertheless, unlike a previous analysis of the same data focusing on the association between stressful life events and depressive symptoms [19] the aim here was not to test specific variables but to reduce the set of variables from the over 30 variables considered (many of which highly intercorrelated) that best describe increased odds of depressive symptoms among a University student population.

## Conclusion

An alarmingly high prevalence of clinical depressive symptoms was observed among Cypriot university students. Identifying the most vulnerable students in need of empowerment and education in health related issues is important in. Of course, there is a wider need to educate this population on how to overcome the stressors which follow academic life, as well as on how to cope with this unique developmental period in the continuity of the individual. Health education and school counseling programs are potentially the most effective ways to empower individuals who are at a higher risk of developing mental health problems. Such programs could help students avoid passive coping strategies and give them the support structures they need to pursue more active strategies. Higher levels of coping flexibility (i.e., high cognitive flexibility, strategy-situation fit, and goal attainment) could lead to higher levels of positive adjustment and lower levels of depressive symptoms [65].
